# Improving Machining Localization and Surface Roughness in Wire Electrochemical Micromachining Using a Rotating Ultrasonic Helix Electrode

**DOI:** 10.3390/mi11070698

**Published:** 2020-07-19

**Authors:** Siying Ling, Minghao Li, Yong Liu, Kan Wang, Yong Jiang

**Affiliations:** 1 Key Laboratory for Precision & Non-traditional Machining of Ministry of Education, Dalian University of Technology, Dalian 116024, China; 2 Key Laboratory for Micro/Nano Technology and System of Liaoning Province, Dalian University of Technology, Dalian 116024, China; 3 Associated Engineering Research Center of Mechanics & Mechatronic Equipment, Shandong University, Weihai 264209, China; 201816477@mail.sdu.edu.cn (M.L.); wangkan@sdu.edu.cn (K.W.); 201736323@mail.sdu.edu.cn (Y.J.)

**Keywords:** wire electrochemical micromachining, ultrasonic-assisted, machining localization, surface roughness, micro gear

## Abstract

Wire electrochemical micromachining (WECMM) technology is regarded a promising method to fabricate high aspect ratio microstructures on hard-to-machining materials, however, the by-product accumulation in the machining gap limits its application. In this paper, a new method called ultrasonic-assisted wire electrochemical micromachining (UA-WECMM) is proposed to improve the machining performance of WECMM. Firstly, a flow-field simulation in the machining gap was carried out; the results showed that the ultrasonic vibration of electrode can remarkably enhance the mass transport in the machining gap and improve the machining condition. Secondly, experiments were performed to confirm the effect of ultrasonic vibration, which illustrated that the vibration with proper amplitude can reduce the slit width and improve the morphology of machined surface. Moreover, the influence of other machining parameters were also discussed. Finally, a T-type micro connector with good surface roughness (*R*a 0.286 μm) was fabricated on a 300-μm-thick 304 stainless steel workpiece and a micro gear (diameter: 3.362 mm; *R*a: 0.271 μm) with an aspect ratio of 7 was fabricated on a 2-mm-thick workpiece. It is proved that the proposed ultrasonic-assisted wire electrochemical micromachining method has considerable potential and broad application prospects.

## 1. Introduction

Microstructures and components are in significant demand nowadays due to the miniaturization of components [1]. Wire electrochemical micromachining (WECMM) technology has both the advantage of electrochemical machining (ECM) and wire cutting, and shows promising potential in fabricating high ratio slits and structures on hard-to-machine materials with no burr wear out or remaining stress [2,3]. However, the universally application of WECMM had been restricted when the workpiece becomes thick, as the by-product that mainly consists of insoluble hydroxide precipitation and hydrogen bubbles is very difficult to discharge in the narrow and deep machining gap and would lead to unsatisfactory machining results, even in short-circuit applications [4].

Considerable research has been conducted with the aim of improving mass transport in WECMM. Shin et al. [5] introduced ultrashort voltage pulses in machining process to fabricate microgrooves and gears on 100-µm-thick stainless-steel plates, and the influence of electrical parameters was investigated. Special electrode-like helical electrodes [6] and cut-edge electrodes [7] were used to improve the machining condition. By the rotation of the electrode, the renewal of the electrolyte in the inter-electrode gap was promoted. The two studies also implicated that the special electrode could reduce the slit width by declining the average current on the side wall. He et al. [8] tested the machining of 10-mm-thick Ti-Al alloy using axial electrolyte flushing, and the feed rate could reach 3.0 μm/s under the flushing of the electrolyte along the wire electrode. Klocke et al. [9] used two twisted rotating wires with axial electrolyte flushing to machine a 40-mm-thick Inconel 718 workpiece with the cutting rate of 20 mm^2^/min. Zeng et al. [10] proposed a monodirectional-traveling wire method. The influence of the wire traveling speed on the machining stability was investigated and a microslit with uniform width was fabricated. Kalaimathi et al. [11] used a travelling-wire electrochemical machining method with ozonated aqueous NaCl as the electrolyte to cut Monel 400 alloys. Xu et al. [12] introduced a pulsating radial electrolyte supply, and a tube electrode with microholes was used to cut thick workpieces. Slits with a length of 10 mm and an average width of 0.903 mm were machined with a feed rate of 6 µm/s in a 30-mm-thick block. Vibration is also a widely used auxiliary method: Fang et al. [13] adopt a ribbed wire electrode with large amplitude vibration to enhance electrolyte renewal and bubble removal in WECMM, and a fir-tree-like turbine-disk tenon with a machined surface roughness of *R*a = 0.682 μm was fabricated on a plate of 5 mm in thickness. The applied vibration amplitude was 7.5 mm and the frequency was 1.5 Hz. Xu et al. [14] used an electrode vibration technique to improve the machining efficiency and quality. The effect of bubble behavior was studied by electric field simulation. A vibration of 5 μm in amplitude and 100 Hz in frequency was applied to fabricate the microsquare columnar tool arrays. Xu et al. [15] used both cathode travelling and anode vibration to enhance mass transport. The influence of cathode travelling, anode vibration and pulse conditions on the machining surface roughness were studied experimentally, and a microcamera with surface roughness of *R*a = 0.063 μm and *R*max = 0.740 μm was fabricated. He et al. [16] investigated a WECMM method using axial vibration-assisted multi-wire electrodes. Fifteen wire electrodes at a maximum feed rate of 5.0 μm/s were used to fabricate X-shape microparts of high surface quality with a roughness of *R*a = 128.0 nm, *R*q = 162.0 nm and *R*max = 1.72 μm. Meng et al. [17] proposed a vibrating carbon nanotube fiber (CNF) as tool electrode; compared with smooth tungsten wire, the high hydrophilic surface of CNF made it more difficult for the product to deposit, and the maximum feed rate was doubled, while the standard deviation of the machined slits was improved. A microgear and a micropropeller blade were fabricated on Ni-based metallic glass. Based on the former research, Meng et al. [18] applied bipolar nanosecond pulses and a helical CNF in vibration-assisted WECMM, and the machining performance was further improved. Jiang et al. [19] proposed a vibration-assisted WECMM method with B_4_C particles added in the electrolyte; the effects of the B_4_C particles and wire vibration were discussed. The experiment showed that the low-frequency vibration effectively removed the electrolytic products deposition on the electrode, B_4_C particles helped in reducing bubble accumulation, and array microgrooves were fabricated.

The above-mentioned methods can indeed help in mass transport in WECMM, however, there is a lack of investigation into high-frequency vibration applied in WECMM. These studies mostly focus on large amplitude vibration with low–high frequency, while the ultrasonic vibration has been proved to provide significant improvement in electrochemical machining processes:

Sebastian [20] found that the cavitation effect declines the electrode potential during the ECM process through a simulation. Yang et al. [21] found that the ultrasonic vibration applied to both the tool electrode and electrolytic cell could improve the mass transport condition in the machining gap. Wang et al. [22] and Goel [23] discovered that the ultrasonic vibration could help in reducing the hole taper and improving the machining efficiency in ECM and the jet electrochemical micro drilling process. Wataru et al. [24] proposed that the horizontal–vertical composite ultrasonic vibration of the electrode shows more advantages in ECM than single direction vibration.

This paper proposed an ultrasonic-assisted wire electrochemical micromachining (UA-WECMM) method; the ultrasonic vibration was applied vertically on a rotating helical tool electrode to improve the machining localization and surface quality of microslits; and a flow field model of machining gap was established to simulate the influence of electrode motion. Comparative experiments were conducted to study the effect of different amplitude; other parameters are also discussed. Finally, a T-type micro connector and a micro gear with a high aspect ratio was fabricated by using optimal parameters.

## 2. Machining Mechanism of UA-WECMM

### 2.1. Machining Principle

**Figure 1:**
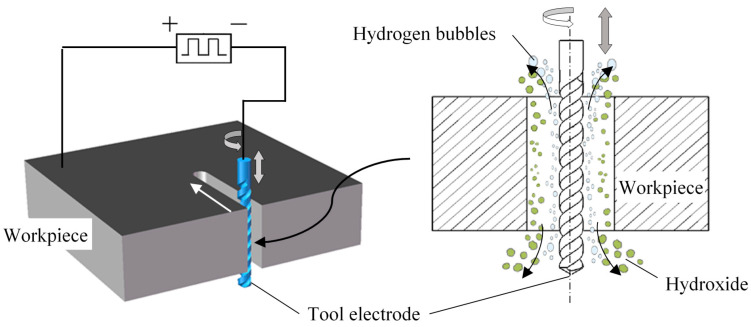
Schematic diagram of the ultrasonic-assisted wire electrochemical micromachining (UA-WECMM) method.

Figure 1 shows the ultrasonic-assisted wire electrochemical micromachining (UA-WECMM) method. The metal workpiece connects the positive pole of an ultrashort pulse power supply to the anode, while the spiral electrode connects the negative one to the cathode. The ultrasonic vibration was applied vertically on the electrode. When the machining process begins, the electrode rotates and feeds along the pre-programed tool path, removing the part in front of the side material under the electrolytic reaction to fabricate structures. Hydrogen bubbles generate on the surface of the electrode and the workpiece dissolves to produce insoluble hydroxide. With the high-frequency axial vibration of the tool electrode, these by-products in the machining gap are discharged efficiently due to the rapid electrolyte velocity alternation and ultrasonic cavitation, ensuring a stable machining process and the desired machining result.

**Figure 2:**
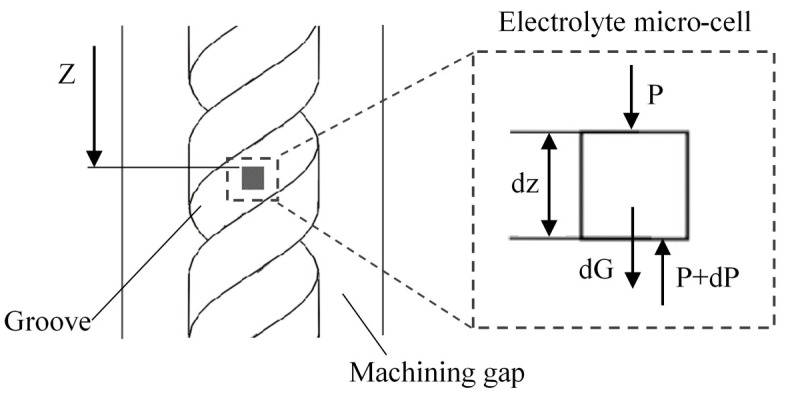
Analysis of the influence of ultrasonic vibration on the electrolyte.

Figure 2 shows the influence of ultrasonic vibration on the electrolyte. An electrolyte microcell is introduced, and the ultrasonic vibration of the tool electrode can be regarded as the sinusoidal movement around the balance position [25]. When the electrode goes up and down, the upper and lower surfaces of grooves push the electrolyte, allowing the vibration of the electrolyte microcell to be expressed as:

(1) z=Asin[ω(t−T)]

where A and ω are the amplitude and frequency of ultrasonic vibration, and T is the time that the ultrasonic wave reaches the microcell. The force acting on the microcell can be expressed as:

(2) PS+dG−(P+dP)S=(dm)a

where P and P+dP are the pressure to the microcell surface on the top and bottom, and S is the cross-sectional area of the microcell. While dG = (dm)g, dm=(dz)Sρ, a is the acceleration of the microcell, which can be expressed as $a=-A\omega^{2}sin[\omega \left(t-T\right)]$, and z is the depth of electrolyte microcell. The Equation (2) can be transferred to:

(3) $$dP=\left\{\rho g+\rho A\omega^{2}sin[\omega \left(t-T\right)]\right\}dz$$

The pressure variation P is:

(4) $$P=\rho gz+\omega^{2}\;A\rho z\;sin\omega \;\left(t-T\right)+P_{0}$$

where ρ is the density of electrolyte, g is the gravitational acceleration, and $P_{0}$ is the atmospheric pressure. The pressure in the interelectrode can be seen to alternate rapidly with the vibration. During the machining process, the transient cavitation generated by the sudden pressure drop can significantly improve the machining condition [26]. The cavitation effect occurs as the negative pressure reaches the cavitation threshold, and small bubbles in the electrolyte are activated to grow and collapse in less than one microsecond, subsequently generating a shock wave and a microjet in the machining gap, which discharges the large hydrogen bubbles and hydroxide from the machining area. The accumulation of by-product is avoided and the stable machining process is ensured. Besides, the periodical growth and collapse of small bubbles raises the interelectrode gas–fluid mixture ratio, which apparently decreases the conductivity in the machining gap and leads to better machining localization. According to the ultrasonic cavitation theory, when the initial bubble diameter, $R_{e}$, is small enough to meet the condition that $P_{1}\ll \frac{2\sigma }{R_{e}}$, the cavitation threshold $P_{B}$ is

(5) $$P_{B}=P_{1}+0.77\frac{\sigma }{R_{e}}$$

where $P_{1}$ is the hydrostatic pressure, and σ is the electrolyte surface tension coefficient. It can be seen from Equation (5) that there is a greater cavitation threshold when the initial bubble diameter is smaller, which means that if the negative pressure in the machining gap becomes greater, the cavitation effect is more extreme due to the activation of more small bubbles. As shown in Equation (4), the interelectrode pressure is notably affected by the amplitude and frequency of vibration, which means that when the frequency is constant, the mass transport in the machining gap is more sufficient and the interelectrode gas–fluid mixture ratio increases in size as the vibration amplitude increases.

### 2.2. Simulation of the Flow Field in the Machining Gap

**Figure 3:**
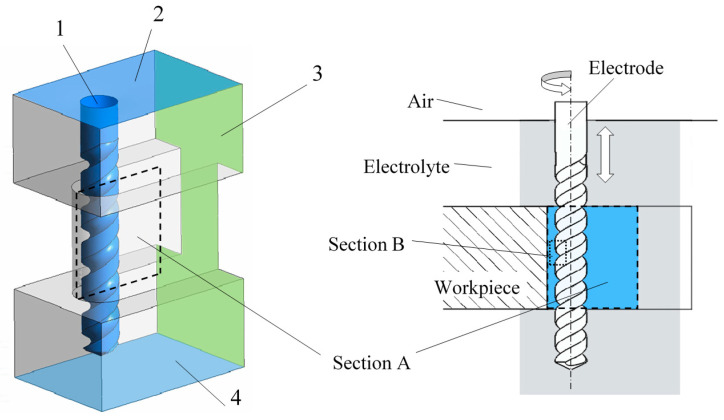
The flow field model of the electrolyte in the machining gap.

A flow field model of an electrolyte was established as shown in Figure 3. The electrolytes in and near the machining gap was taken into consideration for better calculation accuracy. To match the actual size, the slit width was set to 160 μm and the slit height to 300 μm, while the electrode diameter was 100 μm. The simulation was carried out via Fluent 19.0, and the electrolyte was assumed as the constant incompressible Newton fluid. The standard k−ε turbulence model with a proper calculation amount and accuracy was selected. The transport equation is as follows:

(6) $$\frac{\partial \left(\rho k\right)}{\partial t}+\frac{\partial \left(\rho ku_{i}\right)}{\partial x_{i}}=\frac{\partial }{\partial x_{j}}\left[\left(\mu +\frac{\mu_{t}}{\sigma_{k}}\right)\frac{\partial k}{\partial x_{j}}\right]+G_{k}+G_{b}-\rho \varepsilon -Y_{M}$$

(7) $$\frac{\partial \left(\rho \varepsilon \right)}{\partial t}+\frac{\partial \left(\rho \varepsilon u_{i}\right)}{\partial x_{i}}=\frac{\partial }{\partial x_{j}}\left[\left(\mu +\frac{\mu_{t}}{\sigma_{\varepsilon }}\right)\frac{\partial \varepsilon }{\partial x_{j}}\right]+C_{1\varepsilon }\frac{\varepsilon }{k}\left(G_{k}+C_{3\varepsilon }G_{b}\right)-C_{2\varepsilon }\rho \frac{\varepsilon^{2}}{k}$$

The interface of electrode and electrolyte which was marked as Face 1 in Figure 3 was set to the moving wall boundary condition, and an axial ultrasonic vibration with a frequency of 24 kHz and rotation with a rate of 10,000 r/min was applied. Face 3 and 4 were set to the outflow boundary condition. Moreover, the interfaces of air and the electrolyte, i.e., Face 2 and the other faces of the model, were set to the stationary wall boundary condition.

**Figure 4:**
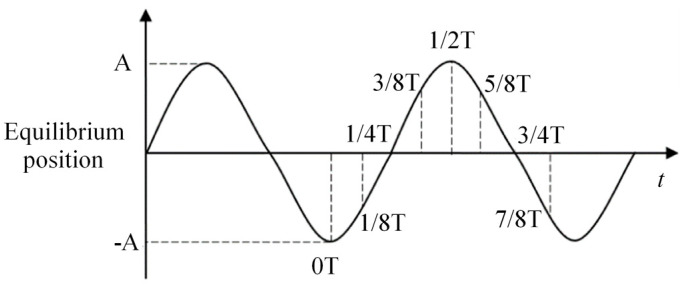
The relative distance between the bottom of the electrode and the workpiece in one period.

The results in Section A and Section B at eight time points as shown in Figure 4 were selected to show the velocity and pressure of the electrolyte in one vibration period. In addition, simulation under the amplitude of 0 μm, 1.4 μm, 2.8 μm and 5.6 μm was carried out to illustrate the influence of vibration amplitude on the flow filed.

**Figure 5:**
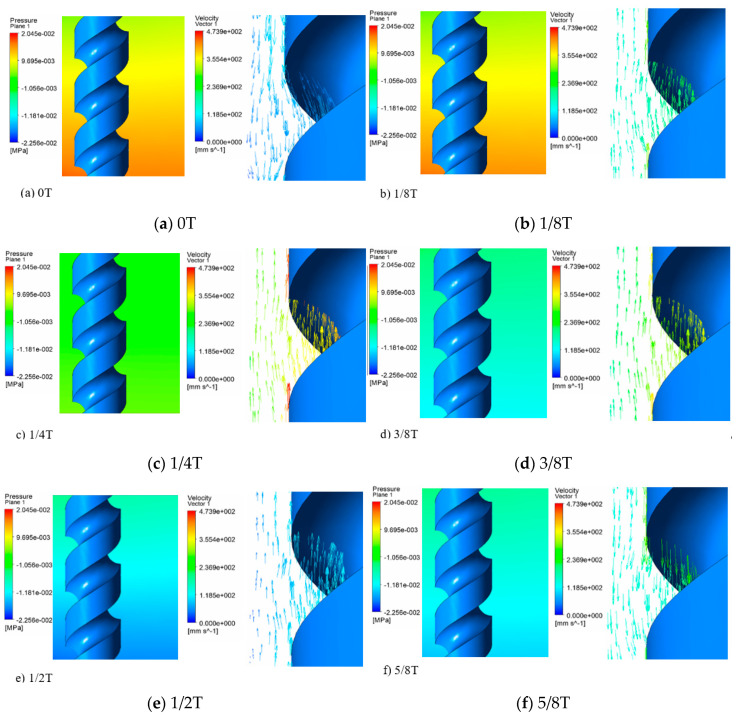

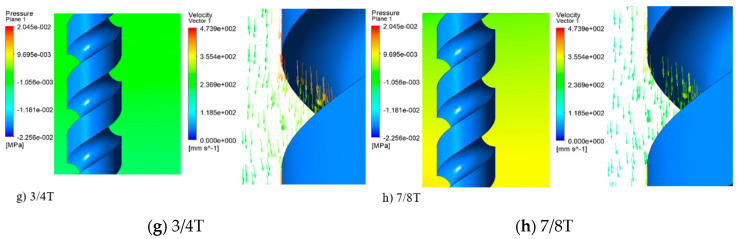
Pressure contours and velocity vector at different times.

Figure 5a shows the state of the flow field when the electrode was at the lowest position. When the former vibration period had just ended, the vibration and rotation were both applied. The electrolyte was found to move downward with low velocity, and the pressure in the machining gap was positive. When the electrode turned upward, as shown in Figure 5b–e, it can be found to have moved upward with remarkable velocity. The hydrogen bubbles produced by the electrolysis reaction were thereby discharged from the machining area to the upper layer of the electrolyte, and the pressure in the machining gap turned negative at the same time and became extreme when the electrode reached the highest position, that is, the cavitation generated in the process. When the electrode turned downward, as shown in Figure 5f–h, the velocity of the electrolyte was inversed and the pressure in the machining gap increased to positive, the cavitation bubbles collapsed, large hydrogen bubbles and insoluble hydroxide were discharged, and a fresh electrolyte was pressed to the machining gap periodically in the process.

**Figure 6:**
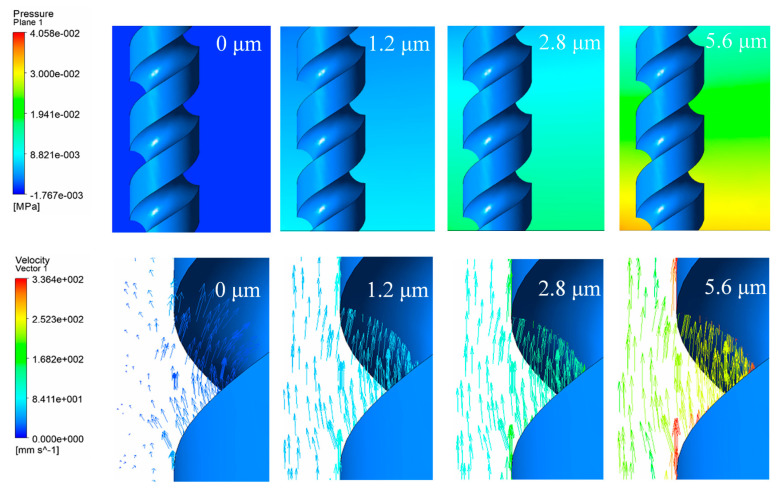
Pressure contours and velocity vector under different amplitude at 1/8T.

A comparison of pressure contours and the velocity vector under different vibration amplitudes is shown in Figure 6. When the vibration amplitude was 0 μm, the electrode rotated with a rate of 10,000 r/min, the pressure in the machining gap was constant, and the electrolyte was found to be stirred by the helical groove. As the vibration amplitude was 1.4 μm, although the electrode continued to rotate, the flow field considerably alternated and the velocity of the electrolyte increased. The pressure in the machining gap also changed. According to the simulations shown in Figure 5, it is clear that the positive pressure at 1/8T corresponds to the negative pressure at 5/8T in one vibration period. Thus, when the amplitude continued to increase, the pressure turned greater and electrolyte velocity increased, indicating more intense mass transport and a larger gas–fluid mixture ratio in the machining gap. It was observed that the vibration of the rotating electrode stirs the electrolyte stronger than with rotation only, and the cavitation effect becomes more significant when amplitude increases, which influences the mass transport and gas–fluid mixture ratio in the machining gap.

## 3. Experimental Setup and Arrangements

**Figure 7:**
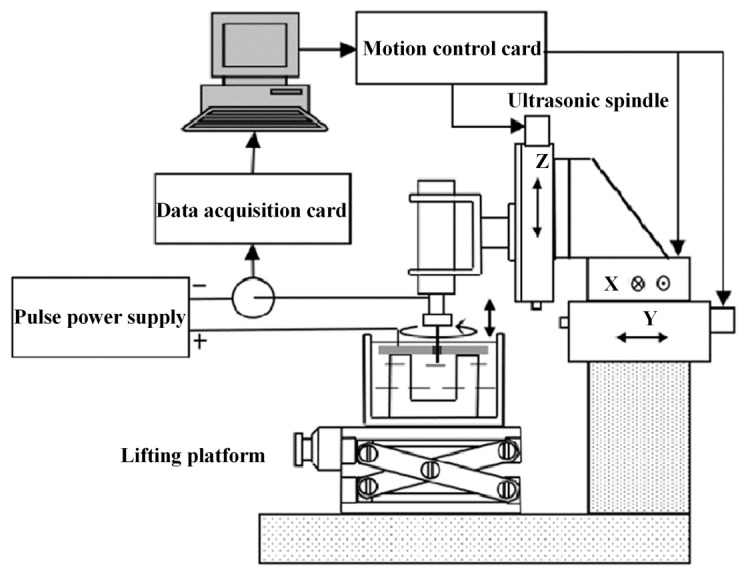
Schematic diagram of the experimental system.

Figure 7 shows the experimental system developed for WECMM with the assistance of ultrasonic vibration. The main components were an X-Y motion stage and a Y motion stage (YA10A-L1, ZA10A-X1T), produced by Kohzu Precision Co., Ltd.,Kawasaki City, Japan; an ultrasonic motorized spindle (YC-USI-D62), produced by Supersonics Technology Co., Ltd., Dongguan City, China; a lifting platform (WN09VM120), produced by Winner Optical Instruments Co., Ltd., Beijing, China; and a pulse power supply (PM8571), produced by Tabor Electronics Ltd., Nesher City, Israel. A helical tungsten electrode with a diameter of 100μm was adopted as the tool electrode, which was fixed on the ultrasonic spindle with an elastic chuck (SK06). The ultrasonic spindle could provide continuous rotation and ultrasonic vibration. The workpiece was fixed by a special fixture in the electrolytic cell. When the machining began, the pulse power supply provided an ultrashort pulse current which was monitored by the detection system during the machining process. The feed rate of the two motion stages and the motion of the motorized spindle was controlled by the control system.

A 304 stainless steel sample with a thickness of 300 μm was ultrasonic-cleaned before being experimented on as the workpiece. Based on the pre-experiment of processing stability, the selected machining parameters are listed in Table 1. After machining, the roughness and topography of the machined surface were measured by a laser scanning confocal microscopy (VK-X1000, KEYENCE Corporation, Osaka City, Japan), and Sa and Ra was obtained. Scanning electron microscopy (Nova NanoSEM450, FEI Company, Hillsborough, Oregon, USA) was used for observing the machined workpiece and measuring the slit width. The data points represent the average values obtained by repeating the experiments at least 3 times.

## 4. Results and Discussion

### 4.1. Influence of Ultrasonic Amplitude on Slit Width and Surface Roughness

**Figure 8:**
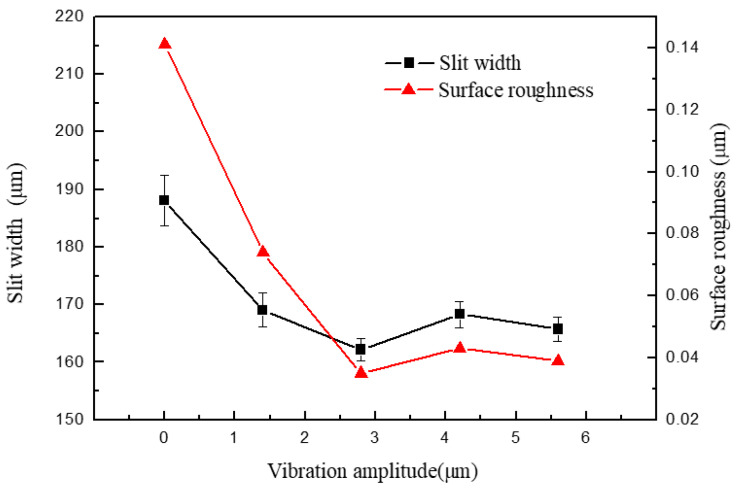
Variations in slit width and surface roughness with vibration amplitude.

Figure 8 shows the width and surface roughness of slits machined by UA-WECMM under different vibration amplitude. The applied voltage was 7 V, the pulse period was 5 μs, the pulse width was 1.5 μs, and feed rate was 1 μm/s. The vibration amplitude was 0 μm, 1.4 μm, 2.8 μm, 4.2 μm and 5.6 μm, respectively.

**Figure 9:**
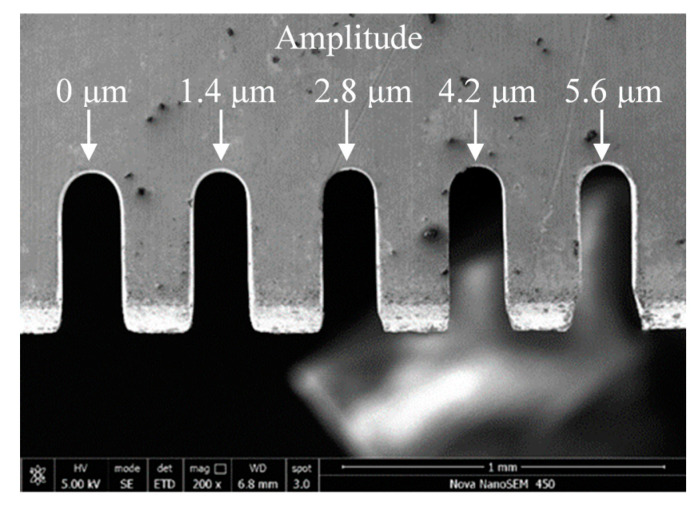
Microslits under different vibration amplitudes.

A clear decrease in slit width can be observed by applying the ultrasonic vibration assistance in the machining process. The slit width was 188.14 μm when the aid of vibration was absent; after adding vibration, the slits narrowed and a 162.22 μm width slit was obtained under an amplitude of 2.8 μm. The increasing amplitude generated greater negative pressure, which caused more small bubbles to grow and collapse, subsequently raising the gas–fluid mixture ratio. The silt width was thereby deceased with lower interelectrode conductivity. However, when the amplitude continued to increase, the slit width began to fluctuate in a limited range. A direct contrast is shown in Figure 9.

**Figure 10:**
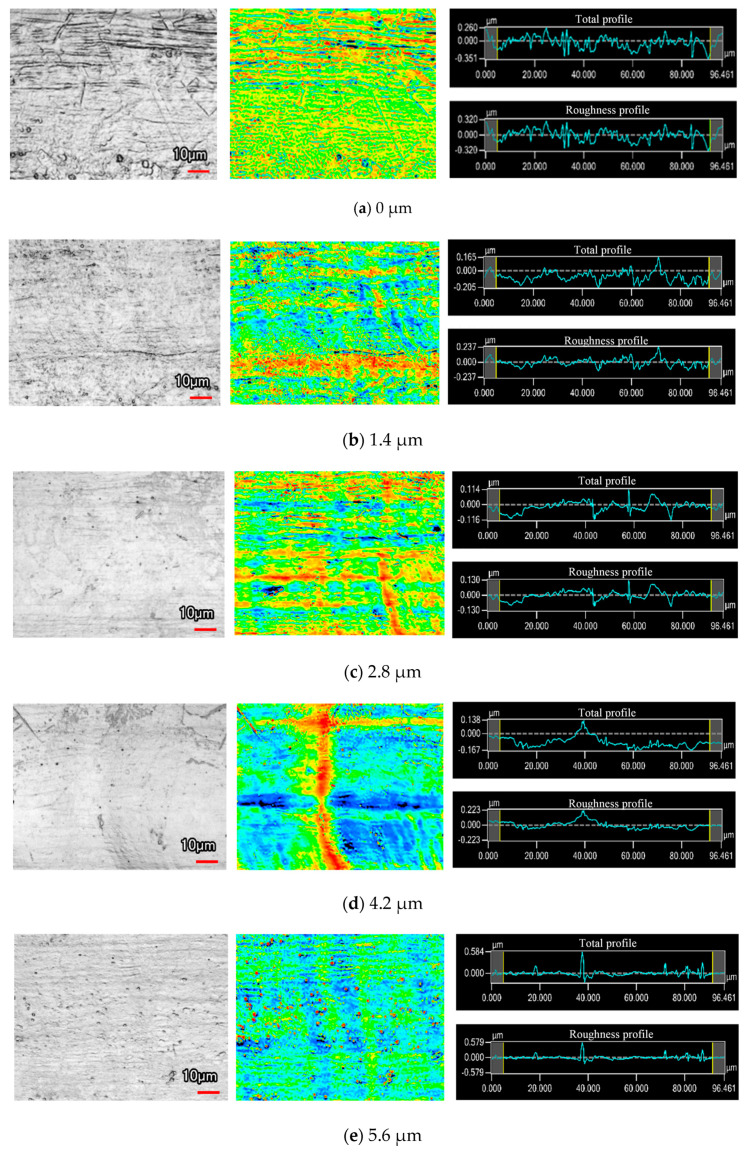
Surface morphology under different vibration amplitudes.

**Figure 11:**
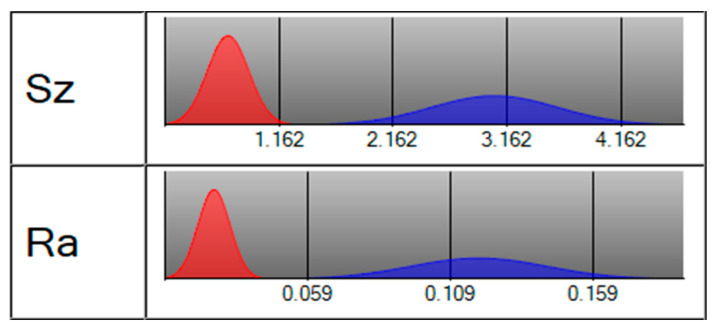
Separation degree of *S*z and *R*a with (red, amplitude of 2.8 μm) and without vibration assistance (blue).

The roughness of the machined surface shows a similar tendency in Figure 8, and the scanning image and measurement result can be seen in Figure 10. When the amplitude was 0 μm, an irregular scratch-like structure could be observed on the slit surface in Figure 10a, which implicates the insufficient mass transport in the interelectrode gap and the distortion of the electric field. The total profile and the roughness profile show continuous oscillation, and the corresponding roughness was 0.141 μm. The surface topography was significantly improved by the application of ultrasonic vibration, as shown in Figure 10b,c. The minimoon roughness of 0.035 μm was obtained under the amplitude of 2.8 μm. It can also be seen from the separation degree in Figure 11 that the application of ultrasonic vibration assistance can greatly reduce the value of *R*a and *S*z. The shock wave and microjet generated by ultrasonic cavitation strongly influenced the electrolyte to push the precipitation and hydric bubbles out of the gap and made a better machining condition, which improved the electric field distribution and the surface roughness. However, when the amplitude continued increasing, the surface roughness began to fluctuate like the slit width; sudden peaks appeared on the total profile and the roughness profile and micropits appeared on the surface, leading to the deterioration of roughness.

**Figure 12:**
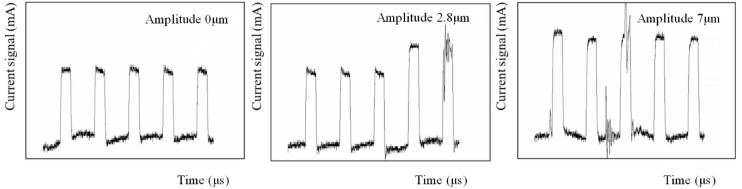
Current waveforms under different vibration amplitudes.

Figure 12 shows the current signal under different amplitudes. When the ultrasonic amplitude was 0 μm, the current waveforms were stable. After applying the vibration, the current waveform gradually altered with an increase in amplitude. This phenomenon implicates the influence of the ultrasonic cavitation and the cause of undesired machining results under large vibration amplitudes. The collapse of the cavitation bubbles could help in mass transport and improve the machining result when the machining gap was wide; however, as the slit width became narrower and the amplitude increased, the conductivity drop caused by the increasing gas–fluid mixture ratio lead to an unstable machining process, thus, the proper amplitude should be selected. The largest slit width was obtained with an amplitude of 0 μm, and it was found that the rotating helical electrode could pump the electrolyte near the helical grooves out of the machining area to some extent, as shown in Figure 6. Thus, even though the machining localization and surface roughness were not ideal, the machining process and the detected current waveform were stable. When the amplitude was 2.8 μm, the current waveform slightly fluctuated, which meant the conductivity decreased due to the raising gas–fluid mixture ratio. The circuit at this point had not yet short circuited, so the machining process could continue for better surface quality and machining localization. As the amplitude increased to 7 μm, extreme oscillations appeared in the current waveform. Subsequently, the gas–fluid mixture ratio became too large, meaning that the conductivity was not able to meet the machining requirement in the narrow machining gap, thereby affecting the slit width and surface roughness. Thus, the amplitude selected should not be too large—based on the experiments and current waveforms, the amplitude of 2.8 μm was selected in the subsequent experiments.

### 4.2. Influence of Voltage on Slit Width

**Figure 13:**
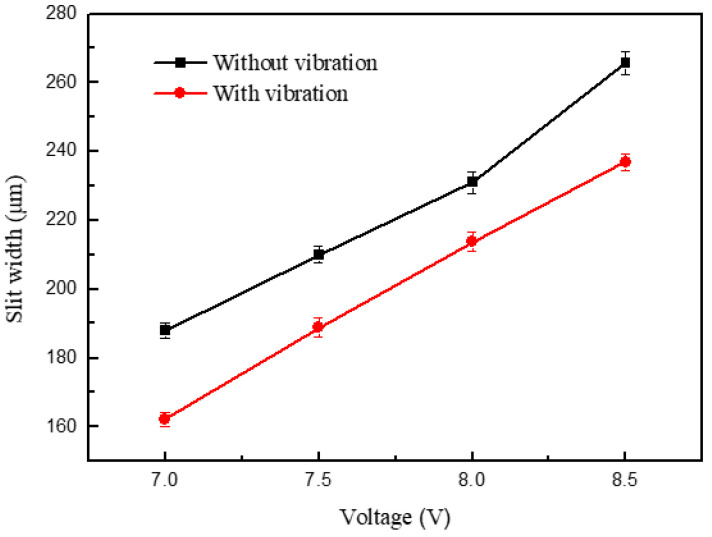
Variation in the slit width in relation to voltage.

**Figure 14:**
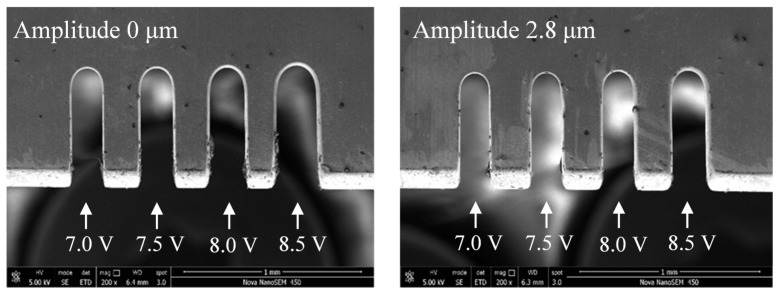
Microslits under different voltages.

Figure 13 shows the variations in the slit width in relation to voltage. The applied voltage was 7.0-8.5 V, the pulse period was 5 μs, the pulse width was 1.5 μs, the feed rate was 1 μm/s, and the vibration amplitude was 2.8 μm. The slit width increased with the increase in voltage. The applied voltage determined the interelectrode electric field—the higher the voltage, the higher the current density. The material removal rate thereby increased, leading to an increase in the slit width and lower machining localization. Apparent contrast in the slit width is shown in Figure 14, while the other parameters remained the same. The slits of the group with the ultrasonic vibration were narrower than those without ultrasonic vibration, which proves the effect of ultrasonic vibration. In both groups of the experiments, when the voltage was less than 7 V, the current density was too small and the erosion of workpiece was insufficient, resulting in unstable machining. Thus, a low voltage is preferred under the promise of stable machining.

### 4.3. Influence of Pulse Parameters on Slit Width

**Figure 15:**
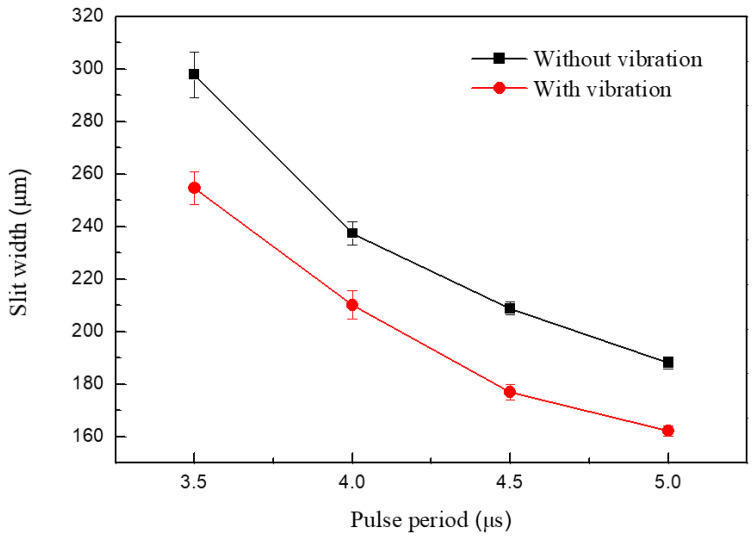
Variations in the slit width in relation to pulse period.

**Figure 16:**
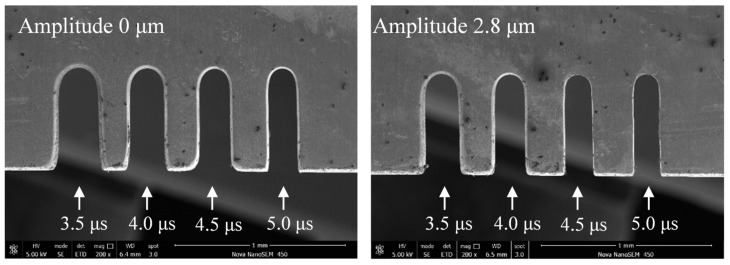
Microslits under different pulse periods.

Figure 15 shows the variation in the slit width in relation to the pulse period. The applied voltage was 7.0 V, the pulse period was 3.5–5.5 μs, the pulse width was 1.5 μs, the feed rate was 1 μm/s, and the vibration amplitude was 2.8 μm. The slit width decreased with the period increase. Slits machined with different pulse periods are shown in Figure 16. When the pulse width was constant, the duty ratio decreased with an increase in pulse period, subsequently causing a drop in the number of pulses per unit of time. The material-moving rate then decreased and the machining localization improved.

**Figure 17:**
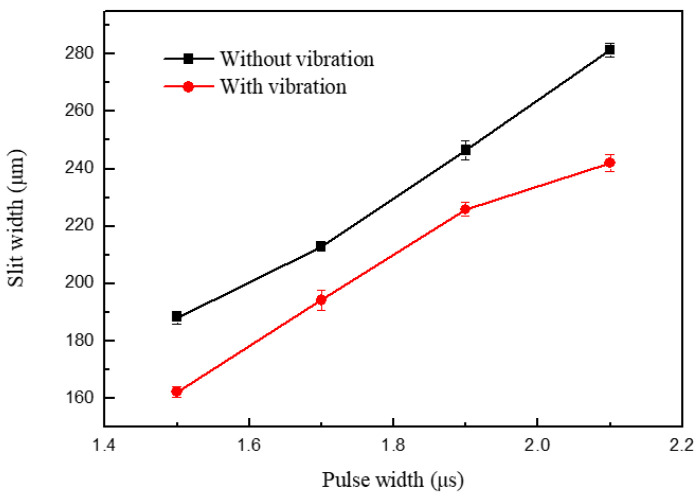
Variation in the slit width in relation to pulse width.

**Figure 18:**
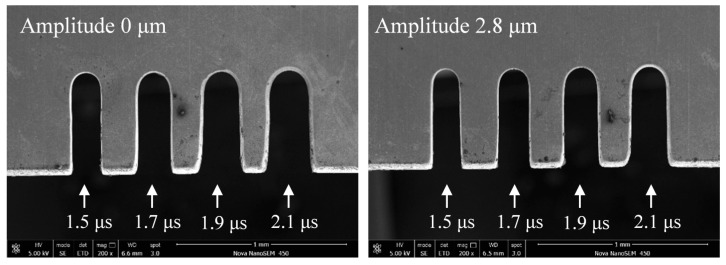
Microslits under different pulse widths.

When the other parameters were constant, the slot width had a pulse period of 5 μs and a pulse width of 1.3–2.1 μs, as shown in Figure 17. Due to a similar reason, the slot width increases with an increase in pulse width; the pulse width determines the effective machining time, which means that the longer the pulse width, the longer the electrolysis reaction time in a single pulse. Thus, the machining localization and the slit edge could be significantly improved in a short pulse width, as shown in Figure 18. The long pulse period and short pulse width also gave time for mass transport to occur by ultrasonic vibration. However, when the two pulse parameters were too extreme, the material-moving rate might be less than the feed rate, and the tool electrode may come into contact with the workpiece and cause a short circuit. Thus, a pulse period of 5 μs and a pulse width of 1.5 μs were chosen as the optimal pulse parameters.

### 4.4. Influence of Feed Rate on Slit Width

**Figure 19:**
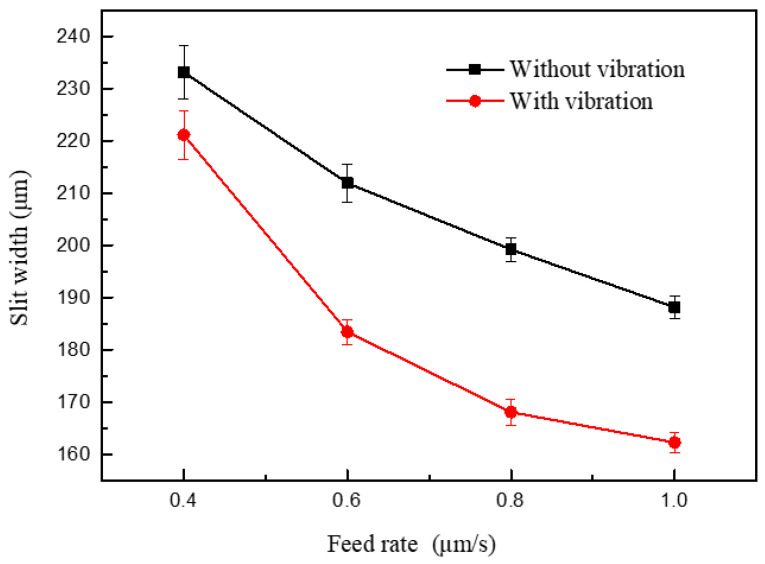
Variation in the slit width in relation to feed rate.

**Figure 20:**
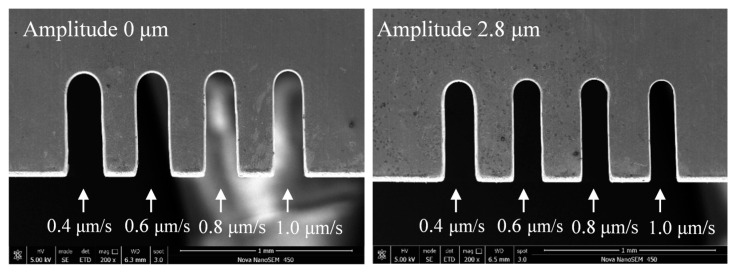
Microslits under different feed rates.

Figure 19 shows the variations in slit width with feed rate. The applied voltage was 7.0 V, the pulse period was 5 μs, the pulse width was 1.5 μs, the feed rate was 0.4 μm/s ~1.2 μm/s, and the vibration amplitude was 2.8 μm. Better slit localization can be obtained when the feed rate is high, while a short current occurs when the electrode feeds at 1.2 μm/s. When other parameters are constant, the higher the feed rate, the less the material corrosion. If the tool electrode remained at the same position for a long time, the electrolytic reaction time between the electrode and workpiece would be too long, and the secondary corrosion on the machined surface would be significant, leading to a larger slit width and a lower surface quality. Thus, in order to improve the machining efficiency and quality, the feed rate should be as large as possible on the premise of ensuring the machining stability. Figure 20 shows the machined slits; the applied feed rate rises from left to right, and the largest feed rate that the electrode with a vibration could work stably is 1 μm/s. The largest slit width was clearly smaller than those in Figure 13 and Figure 17, which means that the feed rate affects does not affect the machining result as much as the electrical parameters. 

### 4.5. Fabrication of MicroStructures

**Figure 21:**
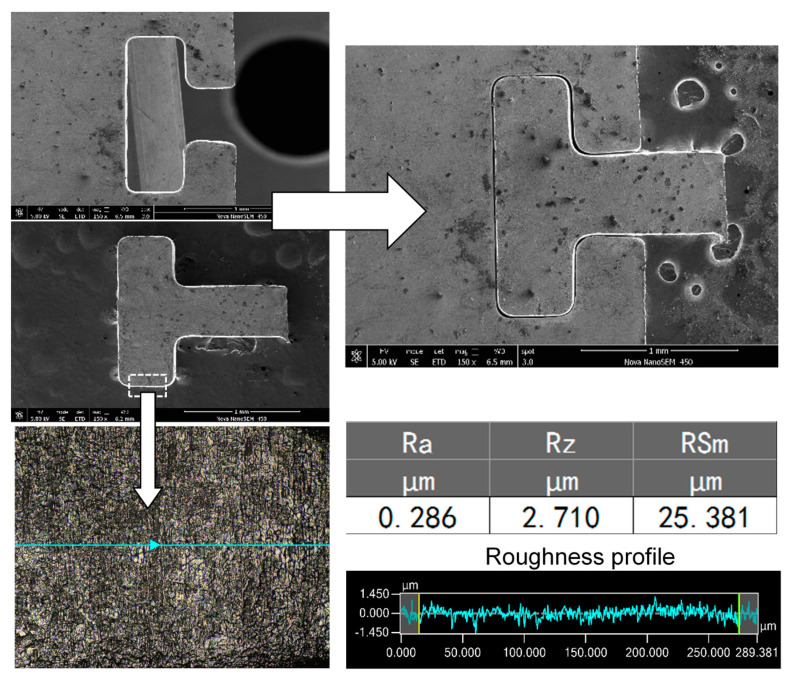
T-type micro connector.

**Figure 22:**
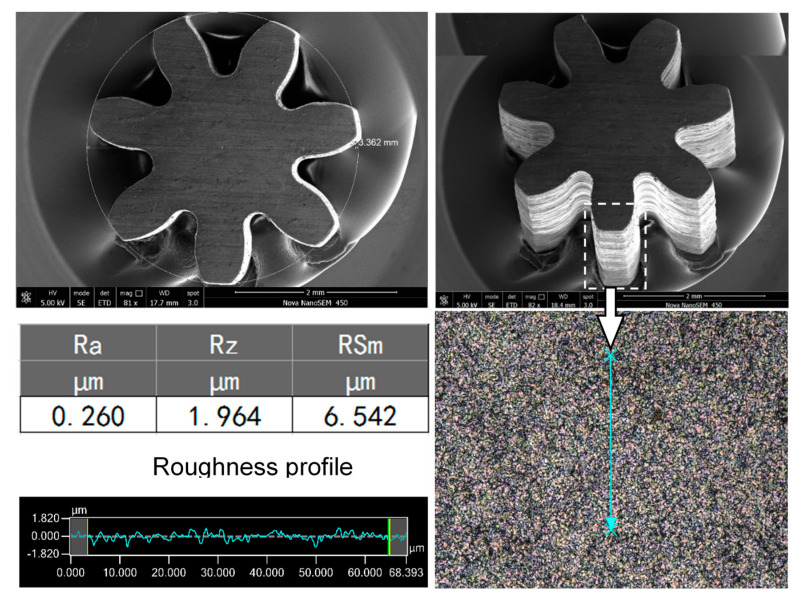
Micro gear with a high aspect ratio.

By using optimized machining parameters—that is, a spindle speed of 10,000 rpm, a sodium nitrate solution at a mass fraction of 5% as the electrolyte, a voltage of 7 V with a pulse period of 5 μs, a pulse width of 1.5 μs, a feed rate of 1.0 μm/s, and an amplitude of 2.8 μm for the ultrasonic vibration—a T-type micro connector with good surface roughness (*R*a 0.286 μm) was fabricated on a 300-μm-thick 304 stainless steel workpiece, as shown in Figure 21, and a micro gear (diameter: 3.362 mm, *R*a: 0.271 μm) was fabricated on a 2-mm-thick workpiece, as shown in Figure 22. The total machining time of the T-type micro connector is about 1.5 h and the machining time of the micro gear is about 4 h.

## 5. Conclusions

The following conclusions can be made in regard to using a UA-WECMM method to improve the localization and surface roughness in WECMM:

(1) The analysis of the electrolyte microcell and simulation of the flow field show that the ultrasonic vibration of the electrode generates a transient pressure alternation which leads to cavitation in the machining gap. Subsequently, large hydrogen bubbles and hydroxide discharge efficiently and the interelectrode gas–fluid mixture ratio increases, which eventually improves the machining quality of the microslit. The increase in vibration amplitude is also observed to enhance the cavitation effect by providing greater negative pressure.

(2) The experiments show that the ultrasonic vibration of the tool electrode can significantly improve the localization and surface roughness of microslits. The detected current signal under different vibration amplitudes implicates that the increase in amplitude in a certain range can improve the machining condition, and when the amplitude becomes too large, the interelectrode gas–fluid mixture ratio severely increases. Thus, the interelectrode conductivity will be too low for stable machining, causing fluctuation in the slit width and surface quality. Other machining parameters have also been discussed and optimized.

(3) By using optimized machining parameters, a T-type micro connector with a good surface roughness (*R*a 0.286 μm) was fabricated on a 300-μm-thick 304 stainless steel plate, and a micro gear (diameter: 3.362 mm, *R*a: 0.271 μm) with an aspect ratio of 7 was fabricated on a 2-mm-thick workpiece. It is proved that the proposed ultrasonic-assisted wire electrochemical micromachining method has considerable potential and broad application prospects.

## Figures and Tables

**Table 1:** Experimental settings.

Parameters	Value
Voltage (V)	7.0, 7.5, 8.0, 8.5
Pulse period (μs)	3.5, 4.0, 4.5, 5.0, 5.5
Pulse width (μs)	1.3, 1.5, 1.7, 1.9, 2.1
Workpiece thickness (μm)	300
Electrode diameter (μm)	100
Electrolyte type	NaNO_3_
Electrolyte mass fraction	5%
Spindle speed (rpm)	10,000
Ultrasonic frequency (Hz)	24k
Ultrasonic amplitude (μm)	0, 1.4, 2.8, 4.2, 5.6
Feed rate (μm/s)	0.4, 0.6, 0.8, 1.0
